# The Rapid Emergence of Tigecycline Resistance in *bla*_KPC–2_ Harboring *Klebsiella pneumoniae*, as Mediated *in Vivo* by Mutation in *tetA* During Tigecycline Treatment

**DOI:** 10.3389/fmicb.2018.00648

**Published:** 2018-04-05

**Authors:** Xiaoxing Du, Fang He, Qiucheng Shi, Feng Zhao, Juan Xu, Ying Fu, Yunsong Yu

**Affiliations:** ^1^Department of Infectious Diseases, Sir Run Run Shaw Hospital, School of Medicine, Zhejiang University, Hangzhou, China; ^2^Department of Clinical Laboratory, Zhejiang Provincial People’s Hospital, People’s Hospital of Hangzhou Medical College, Hangzhou, China; ^3^Department of Clinical Laboratory, Sir Run Run Shaw Hospital, School of Medicine, Zhejiang University, Hangzhou, China; ^4^Institute of Hygiene, Zhejiang Academy of Medical Sciences, Hangzhou, China

**Keywords:** tigecycline resistance, carbapenem-resistant *Klebsiella pneumoniae*, efflux pump, *tetA*, KPC-2

## Abstract

Tigecycline is one of the last resort treatments for carbapenem-resistant *Klebsiella pneumoniae* (CRKP) infections. Tigecycline resistance often occurs during the clinical treatment of CRKP, yet its mechanism has still not been clearly elucidated. This study presents an analysis of a tigecycline resistance mechanism that developed in clinical isolates from a 56-year-old female patient infected with CRKP during tigecycline treatment. Consecutive clonal consistent *K. pneumoniae* isolates were obtained during tigecycline treatment. Whole genome sequencing of the isolates was performed, and putative single nucleotide polymorphisms and insertion and deletion mutations were analyzed in susceptible and resistant isolates. The identified gene of interest was examined through experiments involving transformations and conjugations. Four isolates, two of which were susceptible and two resistant, were collected from the patient. All of the isolates belonged to Sequence Type 11 (ST11) and were classified as extensively drug resistant (XDR). One amino acid substitution S251A in TetA was identified in the tigecycline-resistant isolates. Subsequent transformation experiments confirmed the contribution of the TetA variant (S251A) to tigecycline resistance. The transfer capacity of tigecycline resistance via this mutation was confirmed by conjugation experiments. Using southern blot hybridization and PCR assays, we further proved that the *tetA* gene was located on a transferable plasmid of ca. 65 kb in an *Escherichia coli* EC600 transconjugant. Our results provide direct *in vivo* evidence that evolution in the *tetA* gene can lead to tigecycline treatment failure in CRKP clinical strains that carry *tetA*. Moreover, the transfer capacity of tigecycline resistance mediated by mutated *tetA* is a threat.

## Introduction

Carbapenem-resistant *Klebsiella pneumoniae* (CRKP) infections have emerged worldwide as a serious challenge to public health (Pitout et al., 2015). *K. pneumoniae* resistance to carbapenem is mainly due to the acquisition of carbapenemase, and the carbapenem-hydrolyzing *K. pneumoniae* carbapenemase (KPC) has been the enzyme most commonly identified in *K. pneumoniae* in China (Zhang et al., 2017). *K. pneumoniae* can cause various types of infections, including abdominal and urinary tract infections, liver abscesses, bacteremia, and pneumonia (He et al., 2015). The management of infections caused by CRKP is complicated (Pitout et al., 2015). In fact, carbapenemase-producing *K. pneumoniae* often carry multiple drug resistance genes that enable the strains to exhibit resistance to almost all antibiotics except colistin and tigecycline (da Silva et al., 2012; Pitout et al., 2015).

Tigecycline is one of the last treatment options remaining for carbapenem-resistant *Enterobacteriaceae* (Alhashem et al., 2017; Iovleva and Doi, 2017). A semisynthetic derivative of minocycline, tigecycline is a recent antibiotic that belongs to the glycylcycline class and can overcome the mechanisms of tetracycline resistance (Pankey, 2005). Tigecycline is effective against most carbapenemase-producing bacteria, including *K. pneumoniae*, and has been approved in recent years for clinical use in China (He et al., 2015). However, resistance to tigecycline has emerged since its approval (Hoban et al., 2005). The mechanism for tigecycline resistance has not yet been clearly elucidated. In Gram-negative bacteria, the enhanced expression of resistance–nodulation–cell division (RND)-type efflux pumps was associated with decreased tigecycline susceptibility (Osei Sekyere et al., 2016). Over-expression of the RND efflux pumps, AcrAB and OqxAB, is one of the mechanisms reported most frequently in the *Enterobacteriaceae* species, and several global transcriptional regulators of the AraC family, namely, RamA, MarA, SoxS, and RarA, participate in tigecycline resistance via efflux pump activation (Ruzin et al., 2005; Bratu et al., 2009; Veleba and Schneiders, 2012; Veleba et al., 2012). Nielsen et al. (2014) also reported a new efflux pump operon, *kpgABC*, and identified an insertion element whose presence correlated with the *in vivo* development of tigecycline non-susceptibility in *K. pneumoniae*. The ribosomal S10 protein is also a general target (via the mutation of *rpsJ*) that has been described for its ability to cause decreased tigecycline susceptibility in both Gram-negative and Gram-positive bacteria (Cannatelli et al., 2014; Beabout et al., 2015).

Previous studies have shown that tigecycline can overcome the mechanisms of tetracycline resistance (Pankey, 2005; Jenner et al., 2013). However, Akiyama et al. (2013) found that the *tetA* gene carried by *Salmonella* species, which imparts their tetracycline resistance and also causes reduced susceptibility to tigecycline. Linkevicius et al. (2016) discovered that evolutionary changes in TetA, TetM, and TetX can cause tigecycline resistance in *Escherichia coli* *in vitro*, and they predicted that TetX might become the most problematic future Tet determinant because it did not have adverse effects on resistance toward other tetracyclines. Most recently, Chiu et al. (2017) reported that widespread mutated *tetA* genes are concerning due to the possible dissemination of increased tigecycline resistance in *K. pneumoniae*.

In this study, consecutive clonal consistent *K. pneumoniae* isolates were collected from a 56-year-old female patient with duodenal papillary carcinoma who was undergoing tigecycline treatment. In the early stages of her tigecycline treatment, the isolates were susceptible to tigecycline (MIC = 1 mg/L); 13 days later, we isolated the first resistant strain with a tigecycline MIC of 32 mg/L. To the best of our knowledge, this study is the first report to present consecutive clonal consistent *K. pneumoniae* isolates while they evolved high-level resistance to tigecycline during *in vivo* treatment. Whole genome sequencing (WGS) of these isolates was completed using the Illumina HiSeq X Ten platform, and subsequent bioinformatics analyses were performed to explore the tigecycline resistance mechanism of these isolates.

## Materials and Methods

### The Patient and Isolates

A 56-year-old female patient was diagnosed with duodenal papillary carcinoma and admitted to the hospital for a Whipple operation. Following surgery, the patient contracted an abdominal infection. Initially, the patient received multiple antimicrobial treatments, including cefotaxime, levofloxacin, and imipenem. Isolates were cultured from the patient during her hospitalization. KPC-2 producing extensively drug resistant (XDR) *K. pneumoniae* susceptible only to colistin and tigecycline were isolated from both blood and ascites samples 23 days subsequent to the Whipple surgery. The antibiotic therapeutic strategy was then adjusted to that of intravenous tigecycline (a 100 mg loading dose followed by 50 mg every 12 h). Thirteen days later, the first tigecycline-resistant isolate was detected from an ascites sample (QJJ49) with a tigecycline MIC of 32 mg/L; 19 days later, another tigecycline resistant isolate was detected from a blood sample (QJJ51) with a tigecycline MIC of 32 mg/L. Tigecycline therapy lasted for 19 days, until the resistant phenotype appeared. At the end of the treatment period, the patient expired from multiple organ dysfunction syndrome (MODS).

### Bacterial Isolates

All of the isolates were preliminarily identified using the VITEK 2 system (bioMérieux, France) and were further confirmed by 16S rRNA gene sequencing. Multilocus sequence typing (MLST) of the isolate was analyzed using the BacWGSTdb server with the entire genome sequence (Ruan and Feng, 2016).

### Antimicrobial Susceptibility Test

The MICs of tigecycline and colistin were determined using standard broth microdilution tests with fresh (<12 h) Mueller–Hinton broth (Cation-adjusted, Oxoid LTD, Basingstoke, Hampshire, England). *E. coli* ATCC 25922 was used for quality control (He et al., 2015). The MICs of other antimicrobial agents were determined using the Etest method. All tests were performed according to the guidelines of the Clinical and Laboratory Standards Institute (CLSI). Antimicrobial susceptibility was determined using the breakpoints approved by the CLSI, with the U.S. Food and Drug Administration (FDA) breakpoints used for tigecycline (≤2.0 mg/L, susceptible; 4.0 mg/L, intermediate; and ≥8.0 mg/L, resistant).

### Whole Genome Sequencing and Analysis

Genomic DNA was extracted using a QIAamp DNA MiniKit (Qiagen, Valencia, CA, United States) following the manufacturer’s instructions. The genomes were sequenced using the Illumina HiSeq^TM^ X Ten platform (Illumina, Inc., San Diego, CA, United States) following the paired-end 2 bp × 150 bp protocol. The whole genome sequences were assembled using CLC Genomics Workbench 9.0 software (Qiagen, Valencia, CA, United States). Resistance-related genes were analyzed using ResFinder 3.0 with a 90% threshold for gene identification and a 60% minimum length (Zankari et al., 2012).

The genomic sequence of the initial isolate QJJ29 was used as the reference genome. The reads from the other isolates were mapped against this reference genome using CLC Genomics Workbench 9 software. Basic variants such as single nucleotide polymorphisms (SNPs) and insertion and deletion mutations were predicted by CLC software and the potential mutations were confirmed by PCR and Sanger sequencing with the primers listed in Supplementary Table S1. Potential large fragment insertions were also searched by CLC software, but not verified by PCR and sequencing.

### Transformation and Conjugation Experiment

DNA fragments carrying the wild-type *tetA* and mutated *tetA* genes were amplified from the tigecycline-susceptible isolate QJJ29 and the tigecycline-resistant isolate QJJ51, respectively, with the primers listed in Supplementary Table S1. Following amplification, the amplimer was cloned into plasmid pCR2.1 (Invitrogen, United States) behind the T7 promoter. *E. coli* ATCC 25922 was used for transformation. The effect of the *tetA* mutation on the tigecycline MIC was examined using standard broth microdilution tests.

The transfer capacity of tigecycline resistance was investigated by conjugation experiments (Fu et al., 2012). We used the rifampicin-resistant *E. coli* EC600 as a recipient and the rifampicin-susceptible isolates QJJ29 and QJJ51 as donors. Transconjugants were selected on MH agar plates supplemented with tetracycline (32 mg/L) and rifampicin (800 mg/L). The tigecycline MIC of the transconjugants was examined using standard broth microdilution tests. The *tetA* gene was analyzed in the EC600 transconjugants by PCR and Sanger sequencing with the primers *tetA*-Com-F and *tetA*-Com-R (Supplementary Table S1).

### S1-PFGE and Southern Blot Hybridization

Genomic DNA was digested with the S1 enzyme following the protocol of Barton et al. (1995).

The DNA fragments were transferred to a positively charged nylon membrane (Millipore, United States), hybridized with a DIG-labeled specific probe and detected with an NBT/BCIP color detection kit (Roche, Germany). The hybridization probe was designed to bind within the middle portion of the *tetA* gene and was synthesized using the primers listed in Supplementary Table S1.

### Nucleotide Sequence Accession Numbers

The Whole Genome Shotgun projects for isolates QJJ29 (Accession No. POUY00000000), QJJ36 (Accession No. POWK00000000), QJJ49 (Accession No. POUX00000000), and QJJ51 (Accession No. POWJ00000000) have been deposited at DDBJ/EMBL/GenBank.

## Results

### Isolate Characterizations

Four *K. pneumoniae* isolates were isolated from a patient during tigecycline treatment at Sir Run Run Shaw hospital in Zhejiang, China, in 2016. All of the isolates belong to Sequence Type 11 (ST11) and were classified as XDR. According to Illumina sequencing and subsequent mapping, the mapping percentages among the isolates all exceeded 99.6% (i.e., they are clonally consistent). The MICs of the isolates to different antibiotics are presented in **Table 1**. The initial isolate, QJJ29, was collected from a blood sample on April 23, 2016, the same day tigecycline treatment was initiated. QJJ29 was resistant to carbapenem but susceptible to tigecycline. Six days later, on April 28, 2016, the second isolate, QJJ36, was collected. QJJ36 was also susceptible to tigecycline. Thirteen days after the commencement of tigecycline treatment, on May 5, 2016, the first tigecycline-resistant isolate, QJJ49 with a tigecycline MIC of 32 mg/L, was collected from an ascites sample. On May 11, 2016, the second tigecycline-resistant isolate, QJJ51, was collected from a blood sample. The clinical data of the isolates are listed in **Table 2**.

**Table 1:** MICs of the QJJ29, QJJ36, QJJ49, and QJJ51 isolates to different antibiotics.

Isolate	MIC (mg/L)^a^								
	PM	IP	MP	CI	AK	CO	TC	MC	TGC
QJJ29	≥256	≥32	≥32	≥32	≥256	0.25	128	8	1
QJJ36	≥256	≥32	≥32	≥32	≥256	0.25	128	8	1
QJJ49	≥256	1	1	≥32	≥256	0.25	≥256	≥256	32
QJJ51	≥256	≥32	≥32	≥32	≥256	0.25	≥256	≥256	32

^a^*PM, cefepime; IP, imipenem; MP, meropenem; CI, ciprofloxacin; AK, amikacin; CO, colistin; TC, tetracycline; MC, minocycline; TGC, tigecycline.*

**Table 2:** Mutations in QJJ51, QJJ49, and QJJ36 compared with QJJ29.

Isolate	Collection date	TGC MIC	Days of ongoing TGC treatment	Reference position of the mutation	Gene and product	Nucleotide change	Amino acid change
QJJ29	April 23, 2016 (blood sample)	1 mg/L	TGC treatment started	–	–	–	–
QJJ36	April 28, 2016 (blood sample)	1 mg/L	6	QJJ29 contig 1: 189918	Hypothetical protein	T1373A	L458Q
				QJJ29 contig 19: 47896	tRNA-Leu	C47T	–
				QJJ29 contig 22: 133	Peptidylprolyl isomerase	Insertion 712CTC	Insertion 238L
QJJ49	May 5, 2016	32 mg/L	13	QJJ29 contig 1: 189918	Hypothetical protein	T1373A	L458Q
	(ascites sample)			QJJ29 contig 19: 47896	tRNA-Leu	C47T	–
				QJJ29 contig 22: 133	Peptidylprolyl isomerase	Insertion 712CTC	Insertion 238L
				QJJ29 contig 2: 69019	*malT*: transcriptional regulator MalT	T2483C	L828P
				QJJ29 contig 79: 1641	TetA family tetracycline resistance MFS efflux *Pump*	T751G	S251A
QJJ51	May 11, 2016	32 mg/L	19	QJJ29 contig 1: 189918	Hypothetical protein	T1373A	L458Q
	(blood sample)			QJJ29 contig 19: 47896	tRNA-Leu	C47T	–
				QJJ29 contig 22: 133	Peptidylprolyl isomerase	Insertion 712CTC	Insertion 238L
				QJJ29 contig 2: 69019	*malT*: transcriptional regulator MalT	T2483C	L828P
				QJJ29 contig 79: 1641	TetA family tetracycline resistance MFS efflux *pump*	T751G	S251A
				QJJ29 contig 32: 8939	*abiU*: AIPR protein	Deletion 178–180GAA	Deletion 60E

### Distribution of Resistance Genes

The resistance genes present in the genomes of the isolates are presented in **Table 3**. All isolates harbored multiple resistance genes. We identified the aminoglycoside resistance gene *rmtB*; the beta-lactam resistance genes *bla*_TEM-1B_, *bla*_CTX-M-65_, *bla*_SHV -2_, and *bla*_KPC-2_; the fluoroquinolone resistance gene *qnrS1*; the fosfomycin resistance genes *fosA* and *fosA3*; the lincosamide resistance gene *lnu(A)*; the phenicol resistance gene *catA2*; the sulphonamide resistance gene *sul2*; the trimethoprim resistance gene *dfrA14*; and the tetracycline resistance gene *tetA*. Isolates QJJ51 and QJJ49 had an additional *lnu(A)* gene that was not present in QJJ29 and QJJ36, and isolate QJJ49 lacked the *bla*_KPC-2_ gene and was susceptible to imipenem and meropenem.

**Table 3:** Distribution of resistance genes in isolates QJJ29, QJJ36, QJJ49, and QJJ51.

Antimicrobial pattern	Resistance gene	Isolates			
		QJJ29	QJJ36	QJJ49	QJJ51
Aminoglycoside resistance	*rmtB*	+	+	+	+
Beta-lactam resistance	*bla*_TEM-1B_	+	+	+	+
	*bla*_CTX-M-65_	+	+	+	+
	*bla*_SHV -2_	+	+	+	+
	*bla*_KPC-2_	+	+		+
Fluoroquinolone resistance	*qnrS1*	+	+	+	+
Fosfomycin resistance	*fosA*	+	+	+	+
	*fosA3*	+	+	+	+
Lincosamide resistance	*lnu(A)*			+	+
Phenicol resistance	*catA2*	+	+	+	+
Sulphonamide resistance	*sul2*	+	+	+	+
Trimethoprim resistance	*dfrA14*	+	+	+	+
Tetracycline resistance	*tetA*	+	+	+	+

### Genomic Analysis

According to the mapping analysis, the QJJ36, QJJ49, and QJJ51 isolates all exhibited greater than 99.6% similarity to the reference genome (QJJ29). Three, five, and six mutation sites were confirmed in QJJ36, QJJ49, and QJJ51, respectively (**Table 2**). The three mutations in QJJ36 (QJJ29 contig 1: 189918; QJJ29 contig 19: 47896; and QJJ29 contig 22: 133) were all present in QJJ49 and QJJ51. The tigecycline MIC of QJJ36 was the same as that of QJJ29. One point mutation in *tetA* (QJJ29 contig 79: 1641) was found in both QJJ49 and QJJ51. This mutation (T751G) results in an amino acid substitution (S251A). One, six, and four potential large fragment insertions were found in QJJ36, QJJ49, and QJJ51, respectively. Most of them were located in intergenic region. Only one gene *rmpA* (in isolate QJJ49) was truncated by the insertion (Supplementary Table S2).

### Effects of the *tetA* Mutation on Tigecycline Resistance

The results of the complementation experiment are presented in **Table 4**. When *E. coli* ATCC 25922 was transformed with the mutated *tetA* gene (25922/pCR2.1-*tetA*+), its tigecycline MIC increased from 0.125 to 8 mg/L, its tetracycline MIC increased from 1 to 128 mg/L, and its minocycline MIC increased from 1 to 16 mg/L. When 25922 was transformed with the wild-type *tetA* gene (25922/pCR2.1-*tetA*), its tigecycline MIC increased only from 0.125 to 0.5 mg/L, while its tetracycline MIC increased from 1 to 64 mg/L and its minocycline MIC increased from 1 to 4 mg/L. No MIC change was found in 25922 when it was transformed with the empty pCR2.1 vector (25922/pCR2.1).

**Table 4:** Changes in the tetracycline, minocycline, and tigecycline MICs during the transformation and conjugation experiments.

Strain	Tetracycline	Minocycline	Tigecycline
	MIC (mg/L)	MIC (mg/L)	MIC (mg/L)
25922	1	1	0.125
25922/pCR2.1	1	1	0.125
25922/pCR2.1-*tetA*	64	4	0.5
25922/pCR2.1-*tetA*+	128	16	8
QJJ29 (donor)	128	8	1
QJJ51 (donor)	≥256	≥256	32
EC600 (recipient)	1	1	0.125
EC600 transconjugant of QJJ29	64	4	0.5
EC600 transconjugant of QJJ51	128	16	4

### Conjugation of the Tigecycline Resistance Factor

The transfer capacity of tigecycline resistance was confirmed by a conjugation experiment. The tigecycline MIC increased 32-fold in the EC600 transconjugant of QJJ51 compared with that of the parental strain EC600 (from 0.125 to 4 mg/L); furthermore, the tetracycline MIC increased from 1 to 128 mg/L and the minocycline MIC increased from 1 to 16 mg/L (**Table 4**). Conversely, in the EC600 transconjugant of QJJ29, the tigecycline MIC increased only from 0.125 to 0.5 mg/L while the tetracycline MIC increased from 1 to 64 mg/L and the minocycline MIC increased from 1 to 4 mg/L compared with the parental strain EC600.

### Southern Blot Hybridization of the *tetA* Gene

**FIGURE 1:**
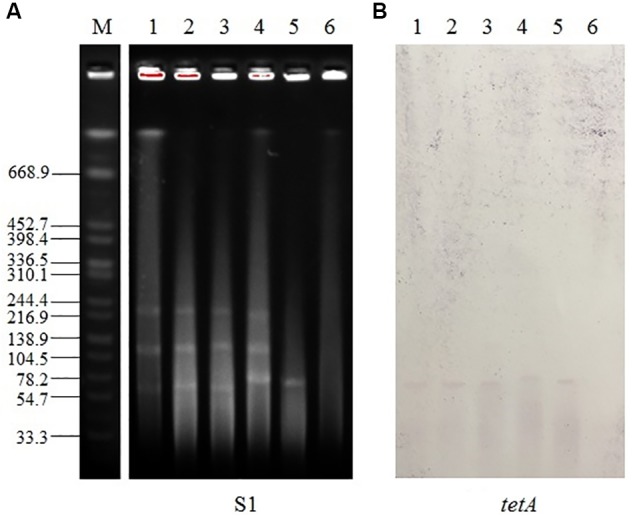
Analysis of *tetA* localization using S1-PFGE and southern blot hybridization. **(A)** S1-PFGE profiles and **(B)** southern blot hybridization with a *tetA* probe. M, *Salmonella enterica* H9812 DNA digested with *XbaI*, used as a molecular marker (kb); lanes 1–6, QJJ29, QJJ36, QJJ49, QJJ51, EC600 transconjugant of QJJ51, and EC600, respectively.

The *tetA* gene was determined to be transferred by conjugation by southern blot hybridization and PCR assays. The *tetA* gene was located on a transferable plasmid with a size of ca. 65 kb in all the isolates detected (**Figure 1**). From the PCR and Sanger sequencing results, the EC600 transconjugant of QJJ51 harbored the same mutant-type *tetA* gene as isolate QJJ51.

## Discussion

In this study, tigecycline resistance was monitored in a 56-year-old female patient infected with CRKP during tigecycline treatment. At the outset of her treatment, the strain was found to be susceptible to tigecycline; 13 days later, the first tigecycline resistant isolate was detected, and it possessed a high tigecycline MIC (32 mg/L).

Over-expression of RND efflux pumps and mutations in the ribosomal S10 protein (*rpsJ*) have been the widely accepted mechanisms that result in decreased tigecycline susceptibility in *K. pneumoniae* (Ruzin et al., 2005, 2008; Bratu et al., 2009; Roy et al., 2013; Villa et al., 2014; Beabout et al., 2015; He et al., 2015). However, the tigecycline resistant isolates (QJJ49 and QJJ51) collected in this study had neither over-expressed RND efflux pumps AcrAB and OqxAB (the expression levels of the efflux pump genes were detected by qRT-PCR, Supplementary Table S3) nor *rpsJ* gene mutations. One mutation in the *tetA* gene (S251A) was uncovered through WGS and analysis. Subsequent transformation and conjugation experiments revealed that the *tetA* mutation was responsible for the development of tigecycline resistance in these isolates.

The wild-type *tetA* gene was observed to slightly decrease the sensitivity to tigecycline in *Salmonella enterica* clinical isolates by Akiyama et al. (2013). In our study, transformation of the wild-type *tetA* gene into *E. coli* ATCC 25922 increased the tigecycline MIC from 0.125 to 0.5 mg/L (**Table 4**). This phenomenon coincides with that of Akiyama’s.

Linkevicius et al. (2016) reported that *tetA* evolution causes decreased sensitivity to tigecycline in *E. coli* *in vitro*. They created libraries of mutagenized *tetA* sequences through separate, error-prone PCR assays, which were then cloned into the pBAD30 vector and electroporated into *E. coli NEB5α*. Subsequently, the researchers selected 53 independent mutants with elevated tigecycline MICs. The most common amino acid substitution identified was G300E. Other frequent changes were identified more than once in the independent mutants including S251A. In another report, Chiu et al. (2017) also frequently isolated mutated *tetA* genes in tigecycline-resistant *K. pneumoniae*.

The current study is the first to provide direct *in vivo* evidence that changes in the *tetA* gene can lead to tigecycline treatment failure in patients infected with CRKP clinical strains. Distinct from Linkevicius’s study, we discovered that the S251A mutation resulted in high-level tigecycline resistance in CRKP strains and *E. coli* ATCC 25922, and their tetracycline and minocycline MICs also increased (**Tables 1**, **4**). Furthermore, our conjugation experiment confirmed the transfer capacity of tigecycline resistance, which was found to be mediated by the mutated *tetA* through a transferable plasmid of ca. 65 kb. Tigecycline is regarded as a last resort treatment for CRKP infections. This finding serves as a therapeutic warning as the *tetA* gene is frequently carried by CRKP strains. Under selective pressure from tigecycline, the *tetA* mutation may occur and lead to treatment failure. This transfer capacity is also a threat to the service life of tigecycline; the more the *tetA* mutates and evolves toward tigecycline resistance, the more likely it will become a horizontally transferrable resistance gene for tigecycline resistance.

## Author Contributions

XD and FH analyzed the genomic data and drafted the manuscript. FH, QS, FZ, JX, and YF performed the experiments. XD and YY designed the study and drafted the manuscript. All authors read and approved the final manuscript.

## Conflict of Interest Statement

The authors declare that the research was conducted in the absence of any commercial or financial relationships that could be construed as a potential conflict of interest.
