# Supplementary material for: The Rapid Emergence of Tigecycline Resistance in blaKPC–2 Harboring Klebsiella pneumoniae, as Mediated in Vivo by Mutation in tetA During Tigecycline Treatment
Source: Front Microbiol. 2018 Apr 5;9:648. doi: 10.3389/fmicb.2018.00648 (PMC5895649; doi:10.3389/fmicb.2018.00648)
Supplement: Supplementary file 1 [file Table_1.DOCX]

**Table S1**. Primers used in this study.

| Primers for mutation confirmation | | |  |
| --- | --- | --- | --- |
| 189918 -F | GCGGATTGCGATGGAT | |  |
| 189918 -R | TTTCGACACGACGGTTCA | |  |
| 47896 -F | AGTTTATGAGGCACGAAGGC | |  |
| 47896 -R | GGAGCTGCGGTGGTTTTA | |  |
| 133 -F | GCGCTTTAGCCTGGTCA | |  |
| 133 -F | CGCCGCAAACGGATACT | |  |
| 69019-MalT-F | GCGTTCGCTGCGTTTG | |  |
| 69019-MalT-R | CGCCGTATCCCATCATCTT | |  |
| 1641-TetA-F | CCACGCTCCGTTCTTCG | |  |
| 1641-TetA-R | TGCCCACCCGTTCCAC | |  |
| 8939-AbiU-F | TGCGTTAGCATTACATTTCAGA | |  |
| 8939-AbiU-R | AACTTTTCAGTGCCTACTTCCA | |  |
| Primers for *tetA* gene transformation (5’-3’) | | |  |
| *tetA-*Com-F | | GTTCCTGAAGTGCCAGTAAAGC |  |
| *tetA-*Com-R | | CGTGCCTCTGCTCACCTTT |  |
| Primers for *tetA* hybridization probe (5’-3’) | | |  |
| 1641-TetA-F | | CCACGCTCCGTTCTTCG | |
| 1641-TetA-R | | TGCCCACCCGTTCCAC | |
