# Supplementary material for: The Rapid Emergence of Tigecycline Resistance in blaKPC–2 Harboring Klebsiella pneumoniae, as Mediated in Vivo by Mutation in tetA During Tigecycline Treatment
Source: Front Microbiol. 2018 Apr 5;9:648. doi: 10.3389/fmicb.2018.00648 (PMC5895649; doi:10.3389/fmicb.2018.00648)
Supplement: Supplementary file 2 [file Table_2.DOCX]

| **Isolate** | **Reference position of the insertion** | **Nucleotide insertion** | **Gene** |
| --- | --- | --- | --- |
| QJJ29 | - | - | - |
| QJJ36 | QJJ29 contig 3: 9917^9918 | CGCAGCTCCGTGCCTGATGC | intergenic region |
| QJJ49 | QJJ29 contig 3: 9917^9918 | CGCAGCTCCGTGCCTGATGC | intergenic region |
|  | QJJ29 contig 12: 30687^30688 | ATGGTGCATCCGGGAGGATTCGAACCTCCGACCGCTCGGTTCGTAGCCGAGTACTCTATCCAGCTGAGCTACGGATGCATCGGGA | intergenic region |
|  | QJJ29 contig 19: 47856^47857 | TGGTGCGAGGGGGGGGACTTGAACCCCCACGTCCGTAAGGACACTAACACCTGAAGCTAGCGCGTCTACCAATTCCGCCACCTTCGC | intergenic region |
|  | QJJ29 contig 29: 44579^44580 | GAAAAAAAGAGAAAAAATAAAAAAAAAGAAAAAAAGAATATGCCGAAAATGTGCACAGAAAAAAAGAGAAAAAATAAAAAAAAAGAAAAAAAGAATATGCCGAAAATGTGCACAG | intergenic region |
|  | QJJ29 contig 41: 5155^5156 | GAAATATGGAAATATGGAAATATGGAAATATGGAAATATGGAAATATGGAAATATGGAAATATGGAAATATGGAAATATGGAAATATGGAAATATGGAAAT | intergenic region |
|  | QJJ29 contig 83: 3970^3971 | TTTTTCAATATTATTTTTTACATTTCCTTGCATGTTAACATAGATGTCATAATCACACCCTTTAGGATAAAACCGCCCCCCCCCC | *rmpA* |
| QJJ51 | QJJ29 contig 3: 9917^9918 | CGCAGCTCCGTGCCTGATGC | intergenic region |
|  | QJJ29 contig 19: 47856^47857 | TGGTGCGAGGGGGGGGACTTGAACCCCCACGTCCGTAAGGACACTAACACCTGAAGCTAGCGCGTCTACCAATTCCGCCACCTTCGC | intergenic region |
|  | QJJ29 contig 21: 104459^104460 | CGCGGGGTGGAGCAGCCTGGTAGCTCGTCGGGCTCATAACCCGAAGGTCGTCGGTTCAAATCCGGCCCCCGCAACCA | intergenic region |
|  | QJJ29 contig 42: 175283^175284 | TTTGGTGGAGCTAAGCGGGATCGAACCGCTGACCTCTTGCATGCCATGCAAGCGCTCTCCCAGCTGAGCTATAGCCCCG | intergenic region |
|  |  |  |  |

**Table S2**. Potential large fragment insertions in QJJ51, QJJ49 and QJJ36 compared with QJJ29.
