# Supplementary material for: The Rapid Emergence of Tigecycline Resistance in blaKPC–2 Harboring Klebsiella pneumoniae, as Mediated in Vivo by Mutation in tetA During Tigecycline Treatment
Source: Front Microbiol. 2018 Apr 5;9:648. doi: 10.3389/fmicb.2018.00648 (PMC5895649; doi:10.3389/fmicb.2018.00648)
Supplement: Supplementary file 3 [file Table_3.DOCX]

**Table S3**. Expression level of *acrA*, *acrB*, *oqxA* and *oqxB* in QJJ29, QJJ36, QJJ49 and QJJ51 compare with reference strain *K. pneumoniae* ATCC13883.

| Isolate | Relative expression ^a^ | | | | MIC  (mg/L) ^b^ |
| --- | --- | --- | --- | --- | --- |
|  | *acrA* | *acrB* | *oqxA* | *oqxB* |  |
| ATCC13883 | 1 | 1 | 1 | 1 | 0.25 |
| QJJ29 | 1.08±0.15 | 0.88±0.19 | ND^c^ | ND^c^ | 1 |
| QJJ36 | 1.58±0.17 | 1.39±0.22 | ND^c^ | ND^c^ | 1 |
| QJJ49 | 1.33±0.32 | 1.06±0.25 | ND^c^ | ND^c^ | 32 |
| QJJ51 | 1.29±0.23 | 1.16±0.12 | ND^c^ | ND^c^ | 32 |

^a^ Relative expression compared with ATCC13883 (expression=1). Results are means of 3 runs ± standard deviation.

^b^ MIC of tigecycline.

^c^ ND, not determined. These four isolates might lack of *oqxA* and *oqxB* genes.
